# Manatee invariants reveal functional pathways in signaling networks

**DOI:** 10.1186/s12918-017-0448-7

**Published:** 2017-07-28

**Authors:** Leonie Amstein, Jörg Ackermann, Jennifer Scheidel, Simone Fulda, Ivan Dikic, Ina Koch

**Affiliations:** 1 Molecular Bioinformatics, Institute of Computer Science, Goethe-University Frankfurt am Main, Robert-Mayer-Straße 11-15, Frankfurt am Main, 60325 Germany; 2 Institute for Experimental Cancer Research in Pediatrics, Goethe-University Frankfurt am Main, Komturstraße 3a, Frankfurt am Main, 60528 Germany; 3 German Cancer Consortium (DKTK), Heidelberg, Germany; 4 German Cancer Research Center (DKFZ), Heidelberg, Germany; 5 Institute of Biochemistry II, Goethe-University Hospital Frankfurt am Main, Theodor-Stern-Kai 7, Frankfurt am Main, 60590 Germany; 6 Buchmann Institute for Molecular Live Sciences, Max-von-Laue-Straße 15, Frankfurt am Main, 60438 Germany

**Keywords:** Signaling pathway, Mathematical model, Petri net, Transition invariant, Feasibility, Manatee invariant, NF- *κ*B pathway

## Abstract

**Background:**

Signal transduction pathways are important cellular processes to maintain the cell’s integrity. Their imbalance can cause severe pathologies. As signal transduction pathways feature complex regulations, they form intertwined networks. Mathematical models aim to capture their regulatory logic and allow an unbiased analysis of robustness and vulnerability of the signaling network. Pathway detection is yet a challenge for the analysis of signaling networks in the field of systems biology. A rigorous mathematical formalism is lacking to identify all possible signal flows in a network model.

**Results:**

In this paper, we introduce the concept of Manatee invariants for the analysis of signal transduction networks. We present an algorithm for the characterization of the combinatorial diversity of signal flows, e.g., from signal reception to cellular response. We demonstrate the concept for a small model of the TNFR1-mediated NF- *κ*B signaling pathway. Manatee invariants reveal all possible signal flows in the network. Further, we show the application of Manatee invariants for in silico knockout experiments. Here, we illustrate the biological relevance of the concept.

**Conclusions:**

The proposed mathematical framework reveals the entire variety of signal flows in models of signaling systems, including cyclic regulations. Thereby, Manatee invariants allow for the analysis of robustness and vulnerability of signaling networks. The application to further analyses such as for in silico knockout was shown. The new framework of Manatee invariants contributes to an advanced examination of signaling systems.

**Electronic supplementary material:**

The online version of this article (doi:10.1186/s12918-017-0448-7) contains supplementary material, which is available to authorized users.

## Background

Living cells interact with their environment to adapt to changes and perturbations. The cell needs to control pivotal decisions such as cell differentiation, proliferation or cell death to provide tissue homeostasis. Signal transduction processes mediate these cellular responses to changes of environmental and intracellular conditions. The appropriate response is orchestrated and processed by a highly intertwined and complex network of signal transduction pathways. Alterations of these processes can result in severe diseases and pathologies [1].

Mathematical models are useful frameworks to capture signaling systems and to gain insights of the system-wide processes. To elucidate and understand the complex regulatory mechanisms of signal transduction networks, various computational models have been applied. The models range from small, but detailed, kinetic models to abstract, large-scale models, which exhibit a coarse-grained view of the cellular processes. Predominantly, ordinary differential equation (ODE)-based kinetic modeling has been applied to quantitatively describe the changes of the species concentrations in time and the resulting substance flow. As the availability of kinetic parameters is often limited, ODE-based modeling is restricted to small and well-characterized processes, such as the I *κ*B-NF- *κ*B signaling module [2]. The majority of experiments on signaling pathways provides mainly qualitative information about the interrelations between the diverse molecular components of a cell. Qualitative data as, e.g., from knockout experiments encourage the application of alternative, topology-based modeling strategies. The behavior of a signaling network can be examined on an abstract level by the application of methods such as Boolean networks [3], interaction graphs [4] and Petri nets (PN) [5]. A Boolean network describes the interdependencies of the entities of a system by a set of Boolean functions. An interaction graph analyzes the network’s topology to reveal the architecture and regulatory principles of the signaling system. The Petri net formalism has been successfully applied to model signal transduction systems, for example, in the human iron homeostasis process [6], in the gene regulation of the Duchenne muscular dystrophy [7], in the Von Hippel-Lindau tumor suppressor interaction network [8] and in the insulin receptor recycling [9, 10].

To investigate the robustness and vulnerability of a signaling network, all possible pathways, i.e., signal flows from signal reception to cellular response, need to be determined. The well-established concept of elementary modes defines sub-processes in metabolic networks that occur under steady-state conditions [11]. Within the PN formalism elementary modes coincide with minimal, semi-positive transition invariants (TI). However, in the analysis of steady-state processes for signaling networks, there are crucial differences that need to be considered for the application of elementary modes or TI. Metabolic systems occur in homeostasis, while signaling pathways display a rather transient and time-dependent behavior. Klamt et al. [4], Behre and Schuster [12] and Schuster and Junker [13] have successfully applied the steady-state assumption to signaling networks as well.

A necessary condition for signal transduction models is to reproduce experimentally measured knockout behaviour. In silico knockout experiments can predict the model behavior to perturbations, such as gene knockouts or inhibitions for drug treatment. A common concept to examine in silico knockouts for mathematical models is based on the computation of TI to reveal correct pathway dependencies. The in silico knockout matrix provides a valuable visualization of the effects of knockouts for signaling systems [14].

Signaling systems exhibit specific characteristics of signal propagation, such as inhibitory relations, crosstalks, feedback loops and signal amplifications. Signal amplification is an important mechanism, since the signal strength is enhanced in signaling cascades. For example, a kinase may have several substrates and thereby catalyzes phosphorylation reactions repeatedly until it is deactivated again. Another pivotal mechanism of signaling is the feedback loop, which influences upstream signaling processes either in a positive or negative way. To gain insights of the biological system, these specific regulatory features need to be captured in the mathematical model. As a consequence, the network topology contains directed cycles that hamper straightforward application of TI for the detection of complete signaling pathways.

To explore the basic behavior and perturbations of signaling systems, many approaches have been proposed to address the challenging issue of pathway analysis. Various adaptations of TI and elementary mode analysis have been introduced. A standard approach for PN is to substitute bidirectional *read arcs* by unidirectional arcs to represent the main direction of the signal flow [15, 16]. Bidirectional arcs represent cycles in the network and thereby affect the TI analysis. The modification simplifies the description of the molecular processes and may ignore important regulatory features. Sackmann et al. [17] have introduced the notion of *feasible* TI to find complete pathways in signaling systems. Feasible TI are specific linear combinations of TI determined via read arcs adjacent to place invariants (PI). The concept of feasible TI is applicable to re-establish a read arc linkage. Nevertheless, an algorithm for the computation of feasible TI is lacking. Behre and Schuster [12] have adapted the concept of elementary flux modes to signaling routes in enzyme cascades, in particular for systems consisting of phosphorylation and dephosphorylation cascades. Klamt et al. [4] have introduced interaction graphs and logical interaction hypergraphs to analyze the structure of signaling and regulatory networks. For interaction graphs, the detection of paths between pairs of species is equivalent to the computation of elementary modes.

Furthermore, alternative, graph-based approaches to analyze signaling pathways have been proposed as well, such as the theoretical framework for detecting signal transfer routes by Zevedei-Oancea and Schuster [18]. This framework applies graph-theoretical breadth-first search to reveal routes in a network from a specific initial factor to a determined target and vice versa. The routes detect dependencies of specific factors on targets. A related approach for signaling networks, which are represented as signed hypergraphs and consider composite nodes, has been presented by Wang and Albert [19]. The introduced method of elementary signaling modes is an extension of the simple path analysis and represents a counterpart to elementary modes. All previous concepts fall short to reveal the complete combinatorial diversity of signal flows at steady state in models of signaling systems, which usually exhibit feedback loops or amplification cycles.

In this article, we adapt the notion of feasible TI [17] and introduce the concept of Manatee invariants (MI) to detect complete pathways from signal reception to cellular response. For an introduction to the terminology of the PN formalism, we refer to the methods section. In the results section, we define the concept of MI to obtain feasibility for TI and give the formal definition of MI. Further, we apply the theoretical framework to the TNFR1-mediated NF- *κ*B pathway and demonstrate the applicability of the concept for in silico knockout experiments. In the supplement, we describe an algorithm for the construction of MI.

## Methods

In this section, we introduce all terms of the Petri net formalism used in the study based on [20–22].

### Petri nets

A Petri net (PN) is a quintuple *N*=(*P*,*T*,*F*,*W*,*m*_0_) with:

- *P* and *T* are finite and disjunct sets of *places* and *transitions*, respectively.
- *F*⊆(*P*×*T*)∪(*T*×*P*) is a set of arcs.
- $W: F \rightarrow \mathbb {N} $ defines the *weight* of each arc.
- $m_{0}: P \rightarrow \mathbb {N}_{0}$ is the *initial marking*.

The directed arcs define *pre-places* and *post-places* for each transition. For a transition *t*∈*T*, the set *F* *t*={*p*∈*P*∣(*p*,*t*)∈*F*} denotes its pre-places and the set *t* *F*={*p*∈*P*∣(*t*,*p*)∈*F*} its post-places. The sets *F* *p*={*t*∈*T*∣(*t*,*p*)∈*F*} and *p* *F*={*t*∈*T*∣(*p*,*t*)∈*F*} define *pre-transitions* and *post-transitions* of a place *p*∈*P*, respectively. A transition is defined as an *output transition* if the transition has only pre-places and no post-places. Analogously, a transition is defined as an *input transition* if it has only post-places and no pre-places.

A *read arc* is a bidirectional arc and denotes a relation of a transition to a pre-place that is also its post-place. A PN is *pure* if no transition exists, for which a pre-place is also a post-place, i.e., without read arcs. A PN is *ordinary* if the weight of each arc is one.

*Tokens* are movable objects, which can be assigned to places. The token distribution is the *marking*, *m*, of a PN and defines a state of the system. The initial state of a system is given by the *initial marking*, *m*_0_. For every state of a system, a place *p* carries an amount of tokens *m*(*p*)≥0. A transition *t*∈*T* has concession, i.e., it is *enabled* or *activated*, in a marking *m* if *m*(*p*)≥*w*(*p*,*t*) applies for each pre-place, *p*∈*F* *t*, and the corresponding weights of the arcs, *w*(*p*,*t*)∈*W*. An enabled transition *t*_*j*_ *fires* by moving tokens from its pre- to post-places according to *m*_*new*_(*p*_*i*_)=*m*_*old*_(*p*_*i*_)+*c*_*ij*_. In the *incidence matrix* *C*, the element *c*_*ij*_ indicates the rearrangement of the tokens on a place *p*_*i*_ if transition *t*_*j*_ fires:

1 $$ c_{ij} := \left\{ \begin{array}{rll} w(t_{j},p_{i}) & &,\text{if}\ p_{i} \in t_{j}F, \\ & -\, w(p_{i},t_{j}) &, \text{if}\ p_{i} \in Ft_{j}, \\ w(t_{j},p_{i}) & -\, w(p_{i},t_{j}) &, \text{if}\ p_{i} \in Ft_{j} \cap t_{j}F, \text{and}\\ & 0 &, \text{otherwise}. \end{array} \right.  $$

#### Feasibility

A firing of a sequence of transitions *σ*=(*t*_1_,*t*_2_,…,*t*_*n*_) results in a shift of tokens $\Delta m:P\to \mathbb {Z}_{0}$ with

$$  \Delta m = C \; x.  $$

The vector $x : T \to \mathbb {N}_{0}$ gives the number of occurrences of transition *t*_*k*_ in the firing sequence *σ* by the component *x*_*k*_=*#* *t*_*k*_. In PN, the vector *x* is the Parikh vector of the sequence *σ* and is denoted by $\overline {\sigma }$.

A sequence of transitions *σ*=(*t*_1_,*t*_2_,…,*t*_*n*_) is called to *originate* from a marking, *m*, if the marking enables the firing of each transition of the sequence. The *p*-th transition, *t*_*p*_, of the sequence, *σ*, must have concession after firing the transitions of the prefix (*t*_1_,*t*_2_,…,*t*_*p*−1_) of sequence *σ*. This condition has to be true for each *p*=1,2,…,*n*. The Parikh vector, $\overline {\sigma }$, of a sequence *σ* that originates from the initial marking *m*_0_ is called *feasible* in the marking *m*_0_. The set *L*_*N*_(*m*) collects all firing sequences that originate from a marking *m* of a PN, *N*. The Parikh vector of each sequence *σ*=*L*_*N*_(*m*) is feasible in the marking *m*. A marking *m* ^′^ is reachable from a marking *m*_0_ if a sequence *σ*∈*L*_*N*_(*m*_0_) exists with $m_{0}\overset {\sigma }{\rightarrow }m'$. A Parikh vector *x* is called *realizable* in the marking *m* if a marking *m* ^′^ is reachable that makes *x* feasible, i.e., a sequence *σ*∈*L*_*N*_(*m* ^′^) exists such that $x=\overline {\sigma }$. A Parikh vector *x* that is not feasible in a marking *m*_0_ may become feasible for a reachable marking *m* ^′^. In this case, *x* is realizable, but not feasible, for the marking *m*_0_. A Parikh vector that is feasible in the marking *m*_0_ is always realizable in the marking *m*_0_ as well.

#### Invariants

A transition invariant (TI) of a PN is defined as a Parikh vector $x:T\to \mathbb {N}_{0}$ that fulfills the equation

2 $$ \Delta m = C \; x = 0.  $$

A TI is denoted as *true* or *semi-positive* if *x* has no negative component, *x*≥0. The set of transitions, whose corresponding components in *x* are positive, is called *support* of *x* and is denoted by *s* *u* *p* *p*(*x*). If the greatest common divisor of its non-null elements is one, a TI is called *canonical*. The set of canonical TI of a PN can be infinite. To obtain finite sets, we introduce the set of *minimal* TI. A TI, *x*, is minimal if no other TI, *x* ^′^, exists such that *s* *u* *p* *p*(*x* ^′^)⊆*s* *u* *p* *p*(*x*). Any TI can be generated from linear combinations of minimal TI. The set of minimal TI is unique and finite. In the following, we consider minimal, semi-positive TI and call them TI. A PN is *covered by TI* (CTI) if each transition occurs in at least one TI. For a Parikh vector *x* of a sequence *σ* that fulfills Eq. (2), we define realizable TI and feasible TI. A Parikh vector *x* is a realizable TI in *N* if a sequence *σ*∈*L*_*N*_(*m* ^′^) exists with $\overline {\sigma } = x$ and a reachable marking *m* ^′^ with $m'\overset {\sigma }{\rightarrow }m'$ [21]. The term feasible TI is a special case of realizable TI [17]. A Parikh vector *x* is a feasible TI in *N* if a sequence *σ*∈*L*_*N*_(*m*_0_) exists with $\overline {\sigma } = x$ and $m_{0}\overset {\sigma }{\rightarrow }m_{0}$.

Equivalently, the equation

3 $$ C^{T} \; y = 0\  $$

defines minimal place invariants (PI). A PI, ${y\in \mathbb {N}_{0}^{|P|}, y \geq 0}$, characterizes a token conservation rule for a set of places. The set of places, whose corresponding components in *y* are positive, is called *support* of *y* and is denoted by *s* *u* *p* *p*(*y*). For further definitions and applications of PN theory, we refer to [5, 20–23] and for algorithms for the computation of invariants and the reachability problem, see [24–26].

## Results and discussion

### Concept of Manatee invariants

**Fig. 1:**
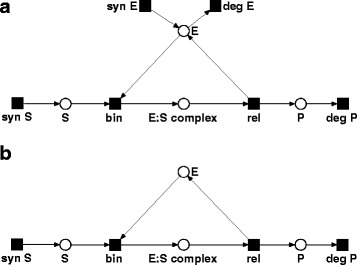
Petri net (PN) of a Michaelis-Menten reaction scheme. **a** The PN describes the synthesis and degradation of an enzyme *E*, which catalyzes the conversion of the substrate *S* to the product *P*. The network has two transition invariants (TI), *T* *I*_1_ =*(syn S, bin, rel, deg P)* and *T* *I*_2_=*(syn E, deg E)*, and therefore is covered by transition invariants (CTI). It has no place invariant (PI). **b** The *T* *I*_1_-induced network, which covers the process of the conversion of *S* to *P*, exhibits a PI, which describes the regaining of the enzyme *E*, either bound in the *E:S complex* or as the free enzyme *E*

Signal transduction systems exhibit cyclic regulations, such as feedback loops, which cause cyclic structures in network models. A common example of a cycle in signaling cascades emerges in amplifications such as enzyme-catalyzed reactions, i.e., reactions that restore the enzyme’s activity. The catalytic reaction describes a recycling of the enzyme and may be represented by a reaction motif like the Michaelis-Menten reaction scheme. Such a recycling step has an impact on the biological relevance of TI. The PN in Fig. 1a describes an enzyme-catalyzed reaction motif. The network is free of PI (PI-free) and CTI, *T* *I*_1_ =*(syn S, bin, rel, deg P)* and *T* *I*_2_=*(syn E, deg E)*. *T* *I*_1_ describes the inflow of substrate *S*, its binding to the enzyme *E*, forming the intermediate *E:S complex* and the production of *P* under release of *E*. *T* *I*_2_ captures the synthesis and degradation of enzyme *E*.

The TI of an enzyme-catalyzed reaction such as in Fig. 1a covers, on the one hand, the recycling of the enzyme, *T* *I*_1_, and, on the other hand, its synthesis and degradation, *T* *I*_2_. Transition *syn E*, which synthesizes enzyme *E*, is not part of *T* *I*_1_ because the coupling of transition *syn E* to *T* *I*_1_ would result in an accumulation of the enzyme. An accumulation contradicts the definition of TI to describe processes at steady state. In signaling systems, synthesis or activation of enzymes is usually regulated by upstream signaling events. The enzyme may need to be activated by upstream processes in the first place, before it is able to catalyze any downstream reactions. Both the upstream processes of enzyme activation and the catalyzed downstream reaction may be covered by TI. However, due to the condition of minimality inherent to TI, the TI that covers the upstream processes and the TI of the downstream reaction remain uncoupled. This is also the case for the small example in Fig. 1a. *T* *I*_2_, which captures the upstream process of the synthesis of *E*, remains uncoupled from the downstream process covered by *T* *I*_1_, which is dependent on *E*.

The small network in Fig. 1a demonstrates an interrelation of TI that cannot be ignored for the analysis of signal transduction networks. Biologically, both TI are interrelated, since the functionality of *T* *I*_1_ depends on the synthesis of *E* in *T* *I*_2_. These findings for TI of enzyme-catalyzed reactions also occur in other cyclic regulations, such as feedback loops. Consequently, for networks with cyclic structures, TI are unable to discover all pathways in terms of sequences of reactions in a network, which correspond to independent processes of signal propagation in a signaling system. The coupling of biologically interrelated TI that represent complete pathways motivated the concept of Manatee invariants (MI).

We adapted the notion of feasible TI of Sackmann et al. [17]. TI that meet the property to be feasible in the initial marking describe an entire biological pathway best assuming that the initial marking corresponds to the physiological state of an unstimulated cell. A TI is feasible in a given input condition or initial marking if all transitions of the TI can fire in sequential order. For a detailed definition of feasibility, see the Methods section. We propose MI as linear combinations of TI to attain feasibility.

The concept of MI is best explained for the small PN in Fig. 1a. The catalyzed conversion of the substrate *S* to the product *P* in *T* *I*_1_ is blocked when no enzyme *E* is present in the system. Consequently, *T* *I*_1_ is only feasible if at least one token is assigned to the place *E* in the initial marking. Alternatively, the enzyme *E* can be produced by firing of transition *syn E* of *T* *I*_2_. The linear combination of *T* *I*_1_ and *T* *I*_2_ is therefore also feasible in the zero initial marking, i.e., no tokens are assigned to any places in the initial marking.

PI have an important impact on the feasibility of TI, since the associated places need to be provided with a sufficient amount of tokens. Let us consider the network in Fig. 1b. This network represents the *T* *I*_1_-induced network of the PN in Fig. 1a. The TI-induced network covers all places and arcs between the transitions of the TI. Whereas the complete network in Fig. 1a is PI-free, the *T* *I*_1_-induced network in Fig. 1b exhibits a PI, *P* *I*_1_=(*E, E:S complex*), which describes the recycling of the enzyme *E*. The linear combination *y*_1_=*T* *I*_1_+*T* *I*_2_ induces a network that matches the network in Fig. 1a and therefore is PI-free.

We define MI as linear combinations of TI that fulfill the following property. The MI-induced network, given by its transitions, all their pre-places and all arcs in-between, is free of PI, except for the PI of the complete PN. We call the MI *pure* if the MI-induced network is PI-free and *impure* if the network contains a PI of the complete PN. Therefore, the pure MI of the PN model in Fig. 1a are *y*_1_=*T* *I*_1_+*T* *I*_2_ and *y*_2_=*T* *I*_2_. Note that, *T* *I*_2_ is feasible in any initial marking, and the *T* *I*_2_-induced network is PI-free.

The construction of MI is motivated by the correlation between the feasibility of TI and the existence of PI in TI-induced networks. We assume a PN with a minimal marking, i.e., tokens are assigned only to places belonging to a PI. For PI within the TI-induced network, no transition of the TI can provide tokens to the places of the PI. Thus, post-transitions of the places of the PI are only enabled if the transitions not belonging to the TI, provide tokens to the places of the PI. Consequently, the TI is not feasible in the minimal initial marking. Therefore, to attain feasibility, the TI need to be coupled to the processes that provide tokens to the PI in the TI-induced network. MI interrelate the corresponding TI and thus constitute a network that is PI-free. Evidently, a PI within the TI-induced network that is simultaneously a PI of the complete PN cannot be dissolved by any coupling to other TI. We assume that PI of the complete PN were assigned with tokens in the initial marking, but any PI within the TI-induced network needs to be provided with tokens by upstream processes of other TI.

### Definition of Manatee invariants

We extended the concept of TI and defined the MI within the PN formalism. We assumed a PN model of the molecular processes of a signal transduction system. These processes should be able to operate in a stable equilibrium. The resources that are required for signal transduction should either be produced by the system itself or have to be provided by the environment. In terms of PN theory, the network should be CTI, pure and PI-free. The following definitions and methods were worthwhile also for other cases, but were motivated by the advantages of their application to PN that are CTI, pure and PI-free.

Definition 1:*TI-induced network*.Let *X*_*TI*_ be the set of TI of the PN, *N*=(*P*,*T*,*F*,*W*,*m*_0_). For *Y*⊆*X*_*TI*_, the TI-induced network is given by *N*_*Y*_=(*P* ^′^,*T* ^′^,*F* ^′^,*W*,*m*_0_) with

- $T' = \bigcup _{x \in Y} supp(x)$,
- $P' = \bigcup _{t \in T'} Ft$ and
- *F* ^′^=((*P* ^′^×*T* ^′^)∪(*T* ^′^×*P* ^′^)) ∩*F*.

The nodes of the TI-induced network, *N*_*Y*_, comprise the transitions of the support, *s* *u* *p* *p*(*x*), of the TI, *x*, in *Y* and all pre-places, *Ft*, of these transitions. The arcs of *N*_*Y*_ are all arcs between the transitions and places in *N*_*Y*_ that are also arcs in *N*.

Definition 2: *PI-free TI-set* (TI-set).For a PN, *N*, let *X*_*TI*_ and *X*_*PI*_ denote the set of TI and the set of PI, respectively. Let *Y*⊆*X*_*TI*_ be a (sub-)set of TI of *N*. *N*_*Y*_ is the TI-induced network of *Y*, and *Y*_*PI*_ denotes the set of PI of *N*_*Y*_. We call the set of TI, *Y*, a *PI-free TI-set* iff

4 $$ \left(\bigcup_{x \in Y_{PI}} supp(x) \right) \ \backslash \ \left(\bigcup_{x \in X_{PI}} supp(x) \right) = \emptyset.  $$

In the following, we will use the term TI-set as a synonym for PI-free TI-set. Apart from the PI of the original PN, the TI-induced network is free of additional PI. For a PN that is CTI, the set of all TI is a TI-set because condition (4) is fulfilled with *Y*_*PI*_=*X*_*PI*_.

Definition 3: *pure/impure* TI-set.Let *Y* be a TI-set of *N*, and *Y*_*PI*_ denotes the set of PI of *N*_*Y*_. We call the TI-set *Y* of *N* *pure*, iff

5 $$ \bigcup_{x \in Y_{PI}} supp(x) = \emptyset\  $$

and *impure* otherwise.

For a PI-free PN, the conditions (4) and (5) become equivalent. The number of TI-sets, *n*, of a given PN is finite with an upper bound of $\phantom {\dot {i}\!}n\le 2^{|X_{TI}|}-1$, where |*X*_*TI*_| is the cardinality of the set of TI of the PN. We introduce the term of a *minimal* TI-set as the minimal basis to construct TI-sets as unions of such minimal TI-sets.

Definition 4: *minimal* TI-set.Let *M* be the set of all TI-sets of a PN. A TI-set, *Y*∈*M*, is *minimal*, iff

$$ \forall A, B \in M,\ A\neq Y, \ B\neq Y : A \cup B \not= Y. $$

Note that, the union of any two TI-sets is a TI-set, and hence, it is sufficient to consider unions of two TI-sets to prove that a minimal TI-set is unique and not a union of a finite number of other TI-sets.

Definition 5: *Manatee invariant* (MI).Let *Y* be a minimal TI-set of a PN. An integer linear combination

$$ y = \sum_{x\in Y}\ c_{x}\ x $$

with $c_{x} \in \mathbb {N}^{+} $ is a Manatee invariant (of *Y*). We call the Manatee invariant, *y*, pure if the TI-set *Y* is pure and impure otherwise.

Because an MI is a linear combination of minimal TI, an MI is not necessarily a minimal invariant. The number of MI is infinite. We define a finite set of MI as *minimal* MI.

Definition 6: *minimal* Manatee invariant.Let *Y* be a minimal TI-set of a PN. An MI, $y =\sum _{x\in Y}\ c_{x}\ x$, is *minimal* if either

- (type a) ∀*x*∈*Y*:*c*_*x*_=1, or
- (type b) *y* is feasible in the initial marking, *m*_0_=0, and no other MI $y' =\sum _{x\in Y}\ c'_{x}\ x$ with *c* ^′^≤*c* is feasible in the initial marking.

Preferably, we would like to compute MI of type b, i.e., MI that are feasible in any initial marking. However, the computation of MI of type b may not be possible because the PN either is not PI-free or contains a motif that restrains the feasibility. Exemplarily, Additional file 1: Figure S2 depicts a pure MI that is not feasible in *m*_0_=0, i.e., the MI is of type a. In the following, we consider only minimal MI. For a description of an algorithm for the construction of MI, we refer to Additional file 1.

### Case study: The NF- *κ*B signaling pathway

**Fig. 2:**
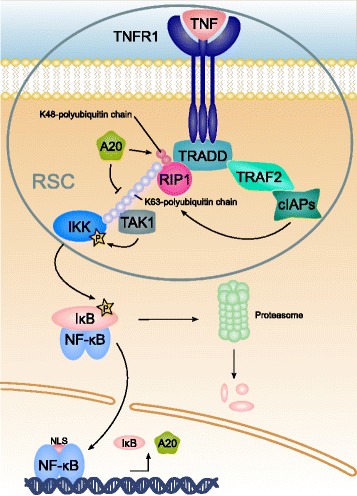
Scheme of TNFR1-mediated NF- *κ*B activation. Following the ligation of cytokine TNF *α* to its receptor TNFR1, the receptor oligomerizes at the membrane and undergoes a conformational change to expose its death domain to the cytosol. Adaptor protein TRADD binds to the death domain and recruits RIP1. TRAF2 binds and recruits in turn E3 ligases, cIAP1 and cIAP2, which catalyze the formation of polyubiquitin chains on RIP1. These polyubiquitin chains serve as a scaffold for the kinases, TAK1 and IKK, to get recruited to the receptor signaling complex (RSC). Organized in proximity, the kinases cross-phosphorylate, and subsequently IKK phosphorylates the inhibitor of NF- *κ*B, I *κ*B, to target it for proteasomal degradation. The transcription factor, NF- *κ*B, is released and translocates into the nucleus due to the exposed nuclear localization signal (NLS). In the nucleus, the transcription factor initiates the gene expression of several target genes. Among them are proteins that regulate NF- *κ*B activation and terminate signal transduction. I *κ*B gets restored in the cytosol, binds the transcription factor in the nucleus to form the inhibitory complex and shuttles it back into the cytosol. A20 is a deubiquitinase, which hydrolyzes the ubiquitin chains and targets RIP1 with K48-linked ubiquitin molecules for proteasomal degradation. The degradation of ubiquitin chains and RIP1 results in the dissociation of the RSC and eventually terminates signal transduction

We applied the theoretical framework of MI to a model of the TNFR1-mediated NF- *κ*B pathway. Figure 2 illustrates the biological processes of the NF- *κ*B pathway. The full protein names are given in the list of abbreviations in the declaration section.

TNFR1 is a membrane receptor, which mediates immune responses triggered by the cytokine TNF *α*. When TNFR1 is activated, various adaptor proteins, TRADD, RIP1, TRAF2 and E3 ligases, cIAP1, cIAP2, are recruited and form the receptor signaling complex (RSC). The E3 ligases catalyze the formation of ubiquitin chains on RIP1 to form a scaffold for the effector kinases, TAK1 and IKK. The activated kinases, TAK1 and IKK, induce the activation of the transcription factor, NF- *κ*B, by targeting the inhibitor of the transcription factor, I *κ*B, for degradation. Released NF- *κ*B can promote gene expression of proinflammatory proteins. The signaling pathway is negatively regulated mainly via two feedback loops that eventually terminate the signal transduction. On the one hand, the inhibitor, I *κ*B, gets restored to terminate NF- *κ*B activity and on the other hand, deubiquitinase A20 promotes the dissociation of the RSC to return to the physiological state of an unstimulated cell. For reviews of the signaling processes, we refer to [27–29].

#### PN model

**Fig. 3:**
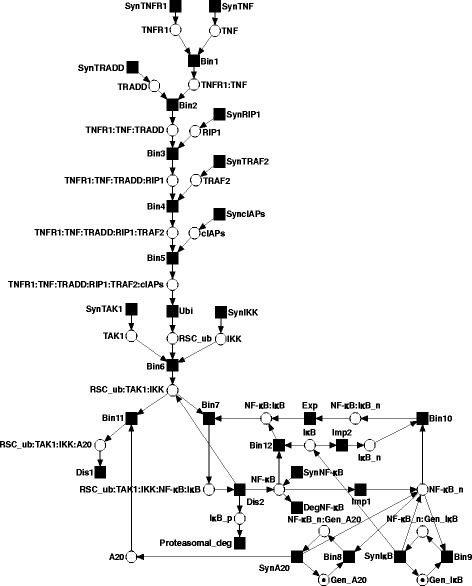
PN of TNFR1-mediated NF- *κ*B signaling capturing the processes depicted in Fig. 2. The PN consists of 31 transitions, 29 places and 69 arcs. The initial marking assigns a token to each of the places, *Gen_A20* and *Gen_I κB*, to provide tokens to the places of the PI of the network (Additional file 1: Table S3)

Figure 3 illustrates the PN model of the NF- *κ*B signaling pathway. The model comprises the main signal flow of receptor activation, RSC formation and transcription factor activation. The regulation of transcription factor activity, along with initiation of gene expression and coordinated termination by feedback loops were also incorporated in the model. As this model serves as a case study, we restricted the model to the internal regulatory processes of the signaling pathway. Crosstalks or alternative cellular responses were not considered. Additional file 1: Tables S1 and S2 list all places and transitions of the PN model and their biological meaning as proteins or protein complexes and reactions, respectively. We developed and analyzed the model applying the open-source software MonaLisa [30]. We provide the file of the PN model in Additional file 2. The MI were computed by the algorithm described in Additional file 1.

The PN is composed of 31 transitions, 29 places and 69 arcs. The PN is pure and ordinary. The initial marking provides a token on each of the places of the gene of A20, *Gen_A20*, and gene of I *κ*B, *Gen_I κB*. Note that, *Gen_A20* and *Gen_I κB* are places belonging to *P* *I*_1_ and *P* *I*_2_, respectively, see Additional file 1: Table S3. The PI concerns parts of gene expression and corresponds to the conservation of the genes of A20 and I *κ*B. The PN has four TI and fulfills the CTI property, see Additional file 1: Table S4.

The trivial *T* *I*_1_ is highlighted blue in Additional file 1: Figure S3. *T* *I*_1_ consists of two transitions describing the process of NF- *κ*B synthesis and degradation. *T* *I*_1_ is feasible in the initial marking, since transition *SynNF- κB* assigns tokens to the place, *NF- κB*, which are consumed by transition *DegNF- κB*. Therefore, all transitions of *T* *I*_1_ are enabled.

*T* *I*_2_ captures the process of TNFR1 activation and RSC formation coupled with gene expression of the deubiquitinase A20 and dissociation of the RSC promoted by A20. Thereby, *T* *I*_2_ describes the processes of the A20 feedback loop. *T* *I*_2_ is highlighted red in Additional file 1: Figure S3. *T* *I*_2_ is not feasible in the initial marking because gene expression of A20 is blocked without transcription factor NF- *κ*B located in the nucleus. The initial marking does not assign any tokens to the place of nuclear NF- *κ*B, *NF- κB_n*, and thus, neither transition *B* *i* *n*8 nor transition *SynA20* can ever be enabled. The *T* *I*_2_-induced network has two PI, *P* *I*_1_ and a PI comprising the places *NF- κB_n* and *NF- κB_n* *:Gen_A20*. Only *P* *I*_1_ of the complete net is provided with tokens in the initial marking.

*T* *I*_3_ captures the processes of the I *κ*B feedback loop with I *κ*B gene expression, binding of I *κ*B to NF- *κ*B and subsequent activation of NF- *κ*B by the RSC complex targeting I *κ*B for degradation. In Additional file 1: Figure S3, *T* *I*_3_ is colored green. *T* *I*_3_ is not feasible in the initial marking because, e.g., the gene expression of I *κ*B is blocked without NF- *κ*B located in the nucleus. Since the place *NF- κB_n* is not provided with any tokens in the initial marking, transitions *B* *i* *n*9 and *S* *y* *n* *I* *κ* *B* are never enabled. The *T* *I*_3_-induced network exhibits four PI, which correspond to the activation and inhibition of NF- *κ*B in the cytosol, the recycling of nuclear NF- *κ*B, the recycling of the gene of I *κ*B during gene expression of I *κ*B and the release of RSC during NF- *κ*B activation, respectively. The initial marking assigns tokens for the gene of I *κ*B, whereas three PI remain insufficiently provided with tokens.

Additional file 1: Figure S4 depicts the PN and highlights *T* *I*_4_. *T* *I*_4_ captures the processes of NF- *κ*B regulation, i.e., the activation of NF- *κ*B by kinases of the RSC complex and I *κ*B degradation along with NF- *κ*B-dependent initiation of the gene expression of I *κ*B, which gets restored in the cytosol and translocates into the nucleus to shuttle NF- *κ*B back into the cytosol. *T* *I*_4_ is not feasible in the initial marking, since, e.g., the activation of NF- *κ*B is dependent on the kinases, TAK1 and IKK, of the RSC, but the place *RSC_ub:TAK1:IKK* carries no tokens in the initial marking. The *T* *I*_4_-induced network has three PI, which correspond to the conservation of NF- *κ*B in the cytosol and the nucleus, the recycling of the gene of I *κ*B and the recycling of the activated RSC, respectively.

All three TI that are not feasible, *T* *I*_2_, *T* *I*_3_ and *T* *I*_4_, exhibit cycles in their induced networks. The cycles emerge from feedback loops or amplifications, such as the amplification during gene expression initiation or the repeated activation of NF- *κ*B by the kinases of the RSC.

#### Manatee invariant analysis

We applied the concept of MI to the NF- *κ*B pathway model. The computation yielded three MI, see Additional file 1: Table S5. The trivial TI of synthesis and degradation of NF- *κ*B, *T* *I*_1_, is the smallest MI, *M* *I*_1_. The *M* *I*_1_-induced network is free of any PI and hence, *M* *I*_1_ is pure and feasible in the initial marking.

*M* *I*_2_ is composed of all four TI, *T* *I*_1_,*T* *I*_2_,*T* *I*_3_ and *T* *I*_4_, and covers the complete PN. *M* *I*_2_ describes the complete TNFR1-mediated NF- *κ*B signaling pathway, from receptor ligation to transcription factor activation and initiation of feedback loops that eventually terminate signal transduction and restore the physiological state of an unstimulated cell. The *M* *I*_2_-induced network has two PI, *P* *I*_1_ and *P* *I*_2_, of the complete PN, hence *M* *I*_3_ is impure and feasible in the initial marking.

*M* *I*_3_ combines the trivial TI, *T* *I*_1_, with two non-trivial TI, *T* *I*_2_ and *T* *I*_4_. Additional file 1: Figure S5 highlights *M* *I*_3_. Aside from the direct binding of I *κ*B to NF- *κ*B in the cytosol (transition *Bin12*), the *M* *I*_2_-induced network describes the complete PN. *M* *I*_3_ covers the processes of TNFR1 stimulation and subsequent formation of RSC, which leads to the activation of NF- *κ*B and NF- *κ*B-dependent gene expression to terminate signal transduction via two feedback loops. Alternatively to the direct formation of the inhibitory complex of NF- *κ*B and I *κ*B in the cytosol, free NF- *κ*B translocates into the nucleus to initiate the gene expression of I *κ*B. Subsequently, restored I *κ*B translocates into the nucleus, binds to NF- *κ*B and shuttles the inhibitory complex into the cytosol. The *M* *I*_2_-induced network has two PI, *P* *I*_1_ and *P* *I*_2_, and therefore, *M* *I*_3_ is impure and feasible in the initial marking.

The MI reflected the complete combinatorial diversity of signal flows of the PN model. *M* *I*_2_ and *M* *I*_3_ cover complete pathways of TNFR1 signal transduction and subsequent signal termination along with restoration of the initial physiological state. The two MI distinguish for the two processes to form the inhibitory complex of NF- *κ*B and I *κ*B and describe distinct, biologically reasonable processes of NF- *κ*B activation. While TI are suitable to determine feedback loops or other cyclic regulatory features in networks, MI are necessary to detect all the pathways, in which these feedback loops are involved.

#### In silico knockouts

**Fig. 4:**
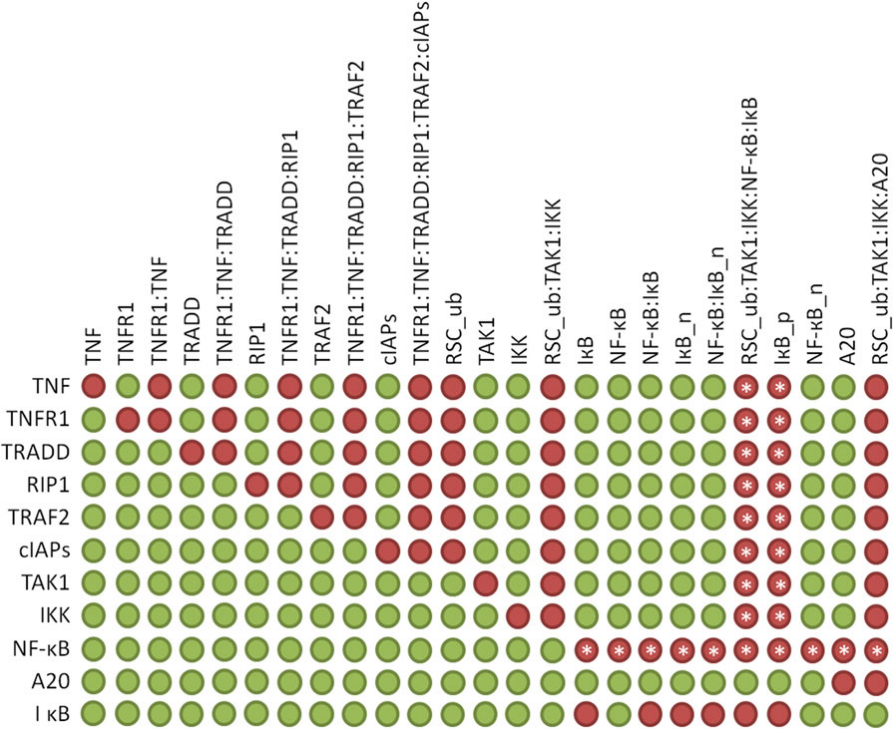
In silico knockout matrix of the PN in Fig. 3 based on Manatee invariants (MI). The rows of the matrix represent a certain protein knockout, while the columns represent the proteins or protein complexes that may be affected by the respective knockout. A *red* entry denotes a negative effect for the protein complex, whereas a *green* entry denotes no effect. The effects that were obtained only for MI-based analysis compared to the TI-based computation are labeled by an asterisk

For further analysis of the NF- *κ*B pathway model, we examined in silico knockouts based on MI. We studied the perturbation of the system for the knockouts of all proteins that constitute the signaling pathway. Figure 4 shows the resulting in silico knockout matrix. To study the influence of a knockout on certain proteins or protein complexes, the in silico knockout analysis adds output transitions to the respective species [14]. Additional file 1: Figure S6 shows the PN model of the NF- *κ*B pathway with the required additional output transitions grayed out. For a detailed description of in silico knockout analysis, we refer to Scheidel et al. [14]. Each row of the matrix represents the knockout of a protein, e.g., by deletion of an input transition that represents the synthesis of the protein. A column represents a protein or protein complex that may be affected by the respective knockout. The entries of the matrix are either green or red. A green entry denotes a place that has a pre-transition in an MI, whereas a red entry denotes a place in the PN that has no pre-transition in an MI. Biologically, a green entry indicates that, e.g., a protein complex can still be formed despite the knockout, while a red entry indicates that the complex cannot be formed.

The rows of the in silico knockout matrix shown in Fig. 4 exhibit two to eleven red entries. The knockout of the deubiquitinase A20 yields two red entries for protein A20 and A20 associated to the RSC complex, *A20* and *RSC_ub:TAK1:IKK:A20*, respectively. All other complexes of the network are unaffected by a knockout of A20, which is in accordance to the known biological behavior, since A20 deubiquitinates the ubiquitin chains within the RSC [31]. The rows of TNF and TNFR1 have the highest number of eleven red entries, followed by TRADD and NF- *κ*B with ten entries each. Therefore, the knockout results indicate a high impact of TNF- *α*, TNFR1, TRADD and NF- *κ*B. The receptor TNFR1 and ligand TNF- *α* have the strongest impact on the system’s behaviour as the pathway is triggered by the receptor binding [32]. The knockout of TRADD, the adaptor protein of the stimulated receptor, has a high impact on the downstream system’s behavior as well. Also NF- *κ*B, the target transcription factor of the signaling pathway, has a strong influence on the functionality of the network. The knockout of NF- *κ*B affects the initiation of the feedback loops of A20 and I *κ*B, while the formation of the RSC is unaffected. In the network, the synthesis of I *κ*B and A20 is dependent on NF- *κ*B activity, therefore, the knockout results match the pathway’s behavior.

The matrix in Fig. 4 is a valuable representation for the effects of knockouts. The knockout results were intuitive and in accordance with expected signaling pathway dependencies. For comprehensive models, the influence of proteins and the detection of dependencies within the pathway may become more complex. In these cases, the in silico knockout can verify whether the model can reproduce experimentally measured knockout behavior, and predictions of the in silico knockout can be tested in further experiments.

The validity of the results of the in silico knockout analysis correlates with the property of MI to describe complete signal flows. The advantage of the application of MI can be conveniently determined in comparison to an in silico knockout analysis based on TI. The asterisks in Fig. 4 indicate red entries that turn green for the corresponding analysis based on TI, see Additional file 1: Figure S7. The in silico knockout matrix based on TI distinguished for 26 entries from the MI-based matrix. Surprisingly, TI-based analysis did not observe any effects for the NF- *κ*B knockout, even NF- *κ*B itself was unaffected by the knockout of its synthesis. The essential role of the target trascription factor, NF- *κ*B, is well known, and many processes in the system are controlled by NF- *κ*B activity [27]. Furthermore, the knockout matrix for TI indicated that the knockout of the proteins that constitute the RSC had no effect on the activation of NF- *κ*B as well, e.g., the knockout of IKK had no effect for the formation of complex *RSC_ub:TAK1:IKK:NF- κB:I κB*. However, the phosphorylation of I *κ*B can be triggered only by the activated kinase, IKK, bound to the ubiquitinated RSC [33]. Therefore in the model, all RSC proteins, including the kinases TAK and IKK, were necessary to initiate the phosphorylation and degradation of I *κ*B. The TI-based analysis was unable to detect these pathway dependencies properly, and the results were misleading concerning their biological interpretation.

For in silico knockouts, the dependencies and relations of pathway components need to be captured correctly. The signal flows of a network reflect these dependencies best. TI or MI that are feasible in the initial marking define signal flows in the network and capture the pathway dependencies most appropriately. For TI that were not feasible, such as *T* *I*_4_, we obtained misleading results for the in silico knockout. *T* *I*_4_ in Additional file 1: Figure S4 covers processes related to NF- *κ*B regulation and exhibits cyclic network structures of feedback loops and amplifications. Considering *T* *I*_4_, a knockout of transition *SynNF- κB* did not affect the TI, and the knockout matrix based on this TI observed no effects for NF- *κ*B knockout. Analogously, *M* *I*_3_ depicted in Additional file 1: Figure S5, comprising *T* *I*_1_, *T* *I*_2_ and *T* *I*_4_, would be affected by the knockout of transition *SynNF- κB*. The valuable concept of MI attains the feasiblility by combination of interrelated TI to capture pathway dependencies of cyclic regulations to upstream or downstream processes. Thereby, only MI were able to derive the correct effects for in silico knockouts.

We proposed the concept of MI as a new method to compute functional pathways in PN models of signaling networks, even for signaling systems with amplification cycles or feedback loops. We showed that the application of MI as a precursor for further analysis like in silico knockouts is beneficial for the investigation of signaling networks. The detection of functional pathways in network models is elementary for many other rigorous network analysis approaches as well. For example, also the examination of crosstalks is dependent on a correct signal flow detection to determine shared processes of different signaling pathways. To evaluate the correctness of a signal transduction model and for a profound investigation that allow to postulate new hypotheses about its dynamic behavior, the computation of all possible signal flows is of great advantage.

Since the concept of MI is based on TI, it takes into account the steady-state assumption and causal relations of the components to determine signal flows in a network model. The proposed algorithm reveals all possible signal flows, which need to be considered for network analysis. An alternative simulation approach would be able to find the identical variety of signal flows in a model at least in the limit of an infinite number of simulation runs. However, the mathematical approach reveals the minimal solutions of all possible signal flows in a model. The mathematical concept of TI is an established precursor for further analyses, such as, e.g., maximal common transition sets [5], minimal cut sets [34] and T-clusters [35], which can be easily applied to MI as well.

A limitation of the rigorous analysis of all pathways in terms of MI is the complexity of the computational task. In worst case, the computation of TI requires exponential space [26]. Since the computation of MI requires the computation of TI, the computation of MI is also at least EXPSPACE-hard. The computation of MI depends on size, structure and complexity of the network and may become infeasible for some network models due to the combinatorial explosion of the search space.

## Conclusions

Characteristic and intrinsic regulation motifs of signal transduction like amplification reactions or feedback loops cause cycles in the topology of a network model. These cycles hamper the straightforward application of TI analysis for the detection of all possible signal flows, since cyclic, minimal TI usually do not reflect the entire pathways. In this article, we introduced the concept of MI, which aims to detect all signal flows from signal reception to cellular response including cyclic regulations. We adapted the concept of feasible TI. MI combine interrelated TI that are disconnected due to cyclic network structures with the objective to attain feasibility. Specific linear combinations of TI interrelate cyclic regulations to linked upstream or downstream processes, reflecting all signal flows from signal reception to cellular response. We presented an algorithm for the construction of MI to compute the combinatorial diversity of pathways from causal dependencies of reactions in a model.

We demonstrated the applicability of the concept of MI for a PN model of the TNFR1-mediated NF- *κ*B signaling pathway. Exemplarily, we elucidated the benefit of MI application for in silico knockout studies. MI-based knockouts revealed correct effects for all protein knockouts of the network, whereas a TI-based analysis failed to detect essential interdependencies of network components. We suggest that other network analysis techniques can also benefit from the concept of MI to obtain biologically relevant conclusions. We presented MI as a straightforward approach for the detection of signal flows to advance modeling and functional pathway analyses, in particular of signal transduction networks.

## Additional files

Additional file 1 Algorithm for the construction of Manatee invariants, Supplementary Figures S2-S7 and Tables S1-S5. (PDF 1116 kb)

Additional file 2 File of the NF- *κ*B Petri net model. (MLPROJECT 68 kb)

## Abbreviations

A20: Tumor necrosis factor *α*-induced protein 3
cIAP1/2: Cellular inhibitor of apoptosis protein 1/2
CTI: Covered by transition invariants
IKK: I *κ*B kinase complex
I*κ*B: Inhibitor of nuclear factor- *κ*B
MI: Manatee invariant
NF- *κ*B: Nuclear factor- *κ*B
NLS: Nuclear localization signal
PI: Place invariant
PN: Petri net
RIP1: Receptor-interacting protein 1
RSC: Receptor signaling complex
TAK1: TGF- *β*-activated kinase 1
TI: Transition invariant
TNF *α*: Tumor necrosis factor- *α*
TNFR1: Tumor necrosis factor receptor 1
TRADD: TNF receptor-associated protein with a death domain
TRAF2: TNF receptor-associated factor 2
Bin: Binding
Deg: Degradation
Dis: Dissociation
Exp: Nuclear export
Imp: Nuclear import
n: Nuclear
p: Phosphorylated
Rel: Release
Syn: Synthesis
Ub: Ubiquitinated
X:Y: Complex of protein X and protein Y
