# Supplementary material for: Manatee invariants reveal functional pathways in signaling networks
Source: BMC Syst Biol. 2017 Jul 28;11:72. doi: 10.1186/s12918-017-0448-7 (PMC5534052; doi:10.1186/s12918-017-0448-7)
Supplement: Supplementary file 1 — Algorithm for the construction of Manatee invariants, Supplementary Figures S2-S7 and Tables S1-S5. (PDF 1116 kb) [file 12918_2017_448_MOESM1_ESM.pdf]

Algorithm for the construction of Manatee invariants

Definitions

Definition 1: *Petri net*

Let $N = (P, T, F, W, m_0)$ be a PN with:

- P and T are finite and disjunct sets of *places* and *transitions*, respectively.
- $F \subseteq (P \times T) \cup (T \times P)$ is a set of arcs.
- $W : F \to \mathbb{N}$ defines the *weight* of each arc.
- $m_0 : P \to \mathbb{N}_0$ is the *initial marking*.

The set of pre-places of a transition t is defined by $Ft = \{p \in P \mid (p, t) \in F\}$. A transition invariant (TI) of a PN with the incidence matrix C is defined as a Parikh vector $x : T \to \mathbb{N}_0$ that fulfills the equation $\Delta m = C\,x = 0$. The set of transitions, whose corresponding components in x are positive, is called *support* of x and is denoted by $supp(x)$. The equation $C^T\ y = 0$ defines minimal place invariants (PI).

Definition 2: *TI-induced network*.

Let X_{TI} be the set of TI of the PN, $N = (P, T, F, W, m_0)$. For $Y \subseteq X_{TI}$, the TI-induced network is given by $N_Y = (P', T', F', W, m_0)$ with

- $T' = \bigcup_{x \in Y} supp(x)$,
- $P' = \bigcup_{t \in T'} Ft$ and
- $F' = ((P' \times T') \cup (T' \times P'))\ \cap F$.

Definition 3:*PI-free TI-set* (TI-set).

For a PN, N, let X_{TI} and X_{PI} denote the set of TI and the set of PI, respectively. Let $Y \subseteq X_{TI}$ be a (sub-)set of TI of N. N_Y is the TI-induced network of Y, and Y_{PI} denotes the set of PI of N_Y. We call the set of TI, Y, a *PI-free TI-set* iff

$$\left(\bigcup_{x \in Y_{PI}} supp(x) \right) \setminus \left(\bigcup_{x \in X_{PI}} supp(x) \right) = \emptyset.$$

Definition 4: *pure/impure* TI-set.

Let Y be a TI-set of N and Y_{PI} denotes the set of PI of N_Y. We call the TI-set Y of N *pure*, iff

$$\bigcup_{x \in Y_{PI}} supp(x) = \emptyset$$

and *impure* otherwise.

Definition 5: *minimal* TI-set.

Let M be the set of all TI-sets of a PN. A TI-set, $Y \in M$, is *minimal*, iff

$$\forall A, B \in M,\ A \neq Y,\ B \neq Y : A \cup B \neq Y.$$

Definition 6: *Manatee invariant* (MI).
Let Y be a minimal TI-set of a PN. An integer linear combination

$$y = \sum_{x \in Y} c_x \ x$$

with $c_x \in \mathbb{N}^+$ is a Manatee invariant (of Y). We call the Manatee invariant, y, pure if the TI-set Y is pure and impure otherwise.

Definition 7: *minimal* Manatee invariants.
Let Y be a minimal TI-set of a PN. An MI, $y = \sum_{x \in Y} \ c_x \ x$, is *minimal* if either

- (type a) $\forall x \in Y : c_x = 1$, or
- (type b) y is feasible in the initial marking, $m_0 = 0$, and no other MI $y' = \sum_{x \in Y} \ c'_x \ x$ with $c' \leq c$ is feasible in the initial marking.

Algorithm
To compute all MI, we propose the following three steps.

1 **Initialize** M, an initial set of candidates of TI-sets.
2 **Construct** two initial tableaus, T_S and T_F.
3 **Extent** tableau T_S until no further extension is possible. Extract M, the set of TI-sets, from the converged tableau T_S. Construct an MI for each TI-set in M.

In the following, we explain the three steps.

1. Initialization
The initialization steps apply standard PN analysis to compute invariants of the PN and the corresponding properties, which will be required for the construction of the two tableaus, T_S and T_F.

1.1 Compute the set of TI, $X_{TI} = \{x_1, x_2, \ldots, x_n\}$.
1.2 Compute the set of PI, X_{PI}.
1.3 Compute the union of the supports of all PI, i.e.,

$$S \leftarrow \bigcup_{x \in X_{PI}} supp(x).$$

1.4 Initialize a set of candidates of TI-sets, $M = \{Y_1, Y_2, \ldots, Y_n\}$, with each candidate, Y_i, containing only a single TI, i.e., $Y_i = \{x_i\}, \ i = 1, 2, \ldots, n$ with $n = |X_{TI}|$.
1.5 For each set $Y \in M$, construct the induced network N_Y. For each induced network, N_Y, compute and store a set of places, S_Y, the *barren area*, applying the following procedure.

Compute the barren area:
1.5.1 Compute the set of PI of N_Y, Y_{PI}.
1.5.2 Compute the union of the supports of all PI in Y_{PI}, i.e.,

$$S_Y \leftarrow \bigcup_{x \in Y_{PI}} supp(x).$$

1.5.3 Eliminate places that are in a PI of N, i.e., $S_Y \leftarrow S_Y \backslash S$.

1.6 For each set $Y \in M$, compute and store a set of places, F_Y, the *aquifer*, applying the following procedure.

Compute the aquifer:

1.6.1 Compute the union of the pre-places, Ft, of all transitions, t, in TI, $x \in Y$, i.e.,

$$F_Y \leftarrow \{p \in P: \ t \in \bigcup_{x \in Y} supp(x), p \in Ft\}.$$

1.6.2 Eliminate places that are in a PI of N, i.e., $F_Y \leftarrow F_Y \backslash S$.

For algorithms to compute TI and PI of a PN, we refer to [1, 2] and literature cited herein. The initialization step determines for each TI, x,

– a barren area given by the set of places S_Y, which have to be provided with a sufficient amount of tokens to make $x \in Y$ feasible and
– an aquifer given by the set of places, F_Y, which can be provided with tokens by $x \in Y$.

We can picture S_Y as a barren area of places that cannot be sufficiently *serviced* by the TI in Y. On the other hand, F_Y may be pictured as the *aquifer*, which is supplied by TI in Y. We may apply the aquifers F_Y to supply the barren area $S_{Y'}$ of a candidate, Y'. Aquifer and barren area determine the conjunction for the construction of Manatee invariants.

2. Construction of initial tableaus

We combine TI, $x \in X_{TI}$, to obtain sets of TI with an empty barren area, i.e., minimal TI-sets. The algorithm starts with M, a set of candidates of TI-sets that contain only a single TI. We construct two tableaus, T_F and T_S. Each tableau has a row for each candidate, $Y_i \in M, \ i = 1, 2, \ldots, |M|$. We start with one candidate for each TI, $x_i \in X_{TI}$, i.e., $Y_i = \{x_i\}$ for $i = 1, 2, \ldots, |X_{TI}|$. The tableaus have two columns for each place plus one column for each TI. The tableau, T_F, has the superstructure

$$T_F = \left[\begin{array}{ccc} F' & S' & I \end{array} \right]. \tag{1}$$

The matrix, I, is the identity matrix of size $|X_{TI}|$. The matrix, $F' = (f_{ij})$, is of size $|X_{TI}| \times |P|$ and is defined by

$$f_{ij} := \begin{cases} 1 & \text{, if } p_j \in F_{Y_i}, \\ 0 & \text{, otherwise.} \end{cases} \tag{2}$$

The matrix, $S' = (s_{ij})$, is of size $|X_{TI}| \times |P|$ and is defined by

$$s_{ij} := \begin{cases} -1 & \text{, if } p_j \in S_{Y_i}, \\ 0 & \text{, otherwise.} \end{cases} \tag{3}$$

The tableau, T_S, has the superstructure

$$T_S = \left[\begin{array}{ccc} S' & F' & I \end{array} \right]. \tag{4}$$

3. Extension of tableau T_S

To set the barren area of the candidates in M to zero, the algorithm iteratively extends each candidate by further TI. We choose an extension scheme that is similar to the classical Fourier-Motzkin elimination algorithm [1] to explore the combinatorial diversity of possible solutions using a breadth-first search.

3.1 Initialize $M_{\text{old}} \leftarrow M$.

3.2 For each place $p \in P$, append all the rows resulting from the addition of one row of T_F to a row of T_S that set the p-th column of T_S to zero, to the tableau T_S.

3.3 In each new row i and for $k = 2|P|+1, \ldots, 2|P|+|X_{TI}|$, set s_{ik} to 1 for $s_{ik} \geq 2$.

3.4 Delete rows of newly generated candidates that are not unique. The candidate of TI-sets of row i is $Y_i = \{x_{k-2|P|} : s_{ik} = 1,\ k = 2|P|+1, \ldots, 2|P|+|X_{TI}|\}$.

3.5 For each new row i, compute the barren area S_{Y_i}. Set the entries, $s_{ik}, k = 1, \ldots, |P|$, of tableau T_S according to S_{Y_i}, i.e., apply equation (3).

3.6 For each new row i, compute the aquifer F_{Y_i}. Set the entries, $s_{ik}, k = |P|+1, \ldots, 2|P|$, of tableau T_S according to F_{Y_i}, i.e., apply equation (2).

3.7 Delete each old row in T_S that has a nonempty barren area.

3.8 Determine the new set of candidates, M_{new}, from the rows of T_S.

3.9 If $M_{\text{new}} \neq M_{\text{old}}$, set $M_{\text{old}} \leftarrow M_{\text{new}}$ and go back to step 3.2, otherwise terminate with $M \leftarrow M_{\text{new}}$.

Each step generates new candidates by adding one TI to the old candidates. Since the total number of candidates is limited by $2^{|X_{TI}|} - 1$, the iteration will eventually terminate with $M_{\text{new}} = M_{\text{old}}$, i.e., at least after the construction of a maximal number of $n = |X_{TI}| - 1$ new generations. The algorithm iteratively explores the entire combinatorial diversity of solutions and generates the complete set of TI-sets.

The set M may contain non-minimal as well as impure TI-sets. To extract the minimal TI-sets, we construct all unions of two TI-sets and mark all TI-sets that are equal to these unions. The deletion of the marked TI-sets gives the set of minimal TI-sets. To distinguish between pure and impure TI-sets, we recompute the barren area for each TI-set, but set $S = \emptyset$. Minimal TI-sets with empty barren area are pure and otherwise impure.

For each minimal TI-set, $Y \in M$, we construct an MI

$$y = \sum_{x \in Y} x$$

of type a. The decision whether an MI, y, is of type b corresponds to the reachability problem $m_0 \xrightarrow{\sigma} m_0$ with $m_0 = 0$ and $y = \overline{\sigma}$. In general, the reachability problem is decidable, but it is at least EXPSPACE-hard [2, 3]. Please note that the decision about an MI's type can be a hard computational task, especially for PN models with weights larger than one, i.e., for non-ordinary PN.

For optimization of computer memory and CPU runtime, it is advantageous to represent the rows of the tableaus by bit patterns and to apply bit operations in the procedures. For simplicity, we abstain to describe the algorithm in terms of bit pattern operations. The application of bit pattern operations is straightforward.

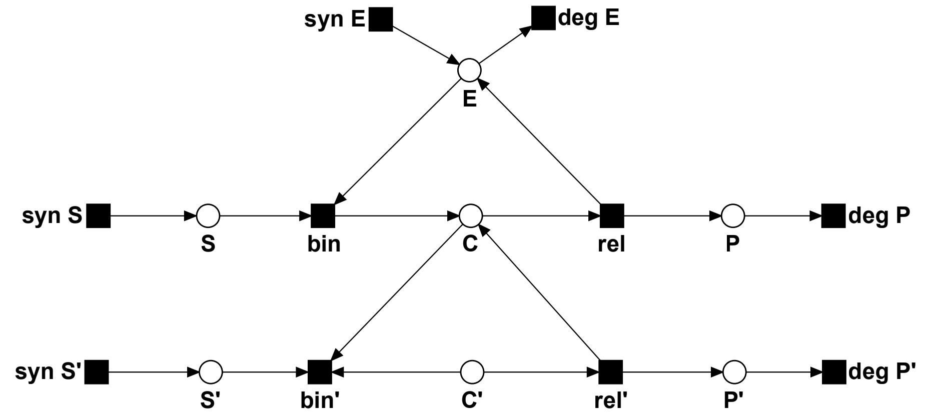

Figure S1 The small network describes the synthesis and degradation of an enzyme E, which catalyzes a second reaction, the conversion of substrate S to product P. The intermediary complex C can catalyze a third reaction of S' to P'. The network is CTI and has no PI. The network has three TI: x_1 = (*syn E, deg E*), x_2 = (*syn S, bin, rel, deg P*), and x_3 = (*syn S', bin', rel', deg P'*). The network has three MI: m_1 = (*syn E, deg E*), m_2 = (*syn E, deg E, syn S, bin, rel, deg P*) and m_3 = (*syn E, deg E, syn S, bin, rel, deg P, syn S', bin', rel', deg P'*).

Example

The PN in Figure S1 has three TI, x_1 = (*syn E, deg E*), x_2 = (*syn S, bin, rel, deg P*), and x_3 = (*syn S', bin', rel', deg P'*). The aquifers are $F_{\{x_1\}} = \{E\}$, $F_{\{x_2\}} = \{E, S, C, P\}$, and $F_{\{x_2\}} = \{C, S', C', P'\}$ and give the matrix (equation (2))

$$F' = \begin{pmatrix} 1 & 0 & 0 & 0 & 0 & 0 & 0 \\ 1 & 1 & 1 & 1 & 0 & 0 & 0 \\ 0 & 0 & 1 & 0 & 1 & 1 & 1 \end{pmatrix}$$

for the order of places (E, S, C, P, S', C', P'). The barren areas are $S_{\{x_1\}} = \emptyset$, $S_{\{x_2\}} = \{E, C\}$, and $S_{\{x_3\}} = \{C, C'\}$ and give the matrix (equation (3))

$$S' = \begin{pmatrix} 0 & 0 & 0 & 0 & 0 & 0 & 0 \\ -1 & 0 & -1 & 0 & 0 & 0 & 0 \\ 0 & 0 & -1 & 0 & 0 & -1 & 0 \end{pmatrix}.$$

In the following, the abbreviated notations $T_S\{x_1, x_2, \ldots, x_n\}$ and $T_F\{x_1, x_2, \ldots, x_n\}$ denote the rows in T_S and T_F, respectively, of a potential TI-set $Y = \{x_1, x_2, \ldots, x_n\}$. The initial tableaus, T_F (equation (1)) and T_S (equation (4)), are

$T_F\{x_1\}$	→	1	0	0	0	0	0	0	0	0	0	0	0	0	0	1	0	0
$T_F\{x_2\}$	→	1	1	1	1	0	0	0	−1	0	−1	0	0	0	0	0	1	0
$T_F\{x_3\}$	→	0	0	1	0	1	1	1	0	0	−1	0	0	−1	0	0	0	1
		↑	↑	↑	↑	↑	↑	↑	↑	↑	↑	↑	↑	↑	↑	↑	↑	↑
		E	S	C	P	S'	C'	P'	E	S	C	P	S'	C'	P'	x_1	x_2	x_3

and

$T_S\{x_1\}$	$\rightarrow$	0	0	0	0	0	0	0	1	0	0	0	0	0	0	1	0	0
$T_S\{x_2\}$	$\rightarrow$	−1	0	−1	0	0	0	0	1	1	1	1	0	0	0	0	1	0
$T_S\{x_3\}$	$\rightarrow$	0	0	−1	0	0	−1	0	0	0	1	0	1	1	1	0	0	1
		↑	↑	↑	↑	↑	↑	↑	↑	↑	↑	↑	↑	↑	↑	↑	↑	↑
		E	S	C	P	S'	C'	P'	E	S	C	P	S'	C'	P'	x_1	x_2	x_3

, respectively. The steps 3.1- 3.7 give the new tableau T_S:

$T_S\{x_1\}$	$\rightarrow$	0	0	0	0	0	0	0	1	0	0	0	0	0	0	1	0	0
$T_S\{x_1, x_2\}$	$\rightarrow$	0	0	0	0	0	0	0	1	1	1	1	0	0	0	1	1	0
$T_S\{x_2, x_3\}$	$\rightarrow$	−1	0	−1	0	0	−1	0	1	1	1	1	1	1	1	0	1	1
		↑	↑	↑	↑	↑	↑	↑	↑	↑	↑	↑	↑	↑	↑	↑	↑	↑
		E	S	C	P	S'	C'	P'	E	S	C	P	S'	C'	P'	x_1	x_2	x_3

The three rows of the tableau correspond to the new candidates of TI-sets $Y_1 = \{x_1\}$, $Y_2 = \{x_1, x_2\}$ and $Y_3 = \{x_2, x_3\}$. The step 3.8 gives a new set of candidates. We now have to repeat the steps 3.2-3.9 until the set of candidates, M_{new}, has converged. The iteration of the steps 3.2-3.9 stops after two repetitions and the final tableau, T_S, reads:

$T_S\{x_1\}$	$\rightarrow$	0	0	0	0	0	0	0	1	0	0	0	0	0	0	1	0	0
$T_S\{x_1, x_2\}$	$\rightarrow$	0	0	0	0	0	0	0	1	1	1	1	0	0	0	1	1	0
$T_S\{x_1, x_2, x_3\}$	$\rightarrow$	0	0	0	0	0	0	0	1	1	1	1	1	1	1	1	1	1
		↑	↑	↑	↑	↑	↑	↑	↑	↑	↑	↑	↑	↑	↑	↑	↑	↑
		E	S	C	P	S'	C'	P'	E	S	C	P	S'	C'	P'	x_1	x_2	x_3

The tableau T_S describes three candidates, $Y_1 = \{x_1\}$, $Y_2 = \{x_1, x_2\}$ and $Y_3 = \{x_1, x_2, x_3\}$. Each of these candidates are TI-sets that are not further extendable. The three TI-sets are pure and minimal. Y_1, Y_2 and Y_3 correspond to three minimal and pure MI of type a, i.e., to $m_1 = x_1 =$ (*syn E, deg E*), $m_2 = x_1 + x_2 =$ (*syn E, deg E, syn S, bin, rel, deg P*) and $m_3 = x_1 + x_2 + x_3 =$ (*syn E, deg E, syn S, bin, rel, deg P, syn S', bin', rel', deg P'*), respectively. The MI, m_1, m_2 and m_3, are also of type b because the corresponding firing sequences that originate from the initial marking, $m_0 = 0$, exist: σ_1=(*syn E, deg E*), σ_2=(*syn E, syn S, bin, rel, deg P, deg E*) and σ_3=(*syn E, syn S, bin, synS', bin', rel', deg P', rel, deg P, deg E*).

References

1. Colom, J.M., Silva, M.: Convex geometry and semiflows in P/T nets. A comparative study of algorithms for computation of minimal p-semiflows. LNCS **483**, 78–112 (1991)
2. Ackermann, J., Einloft, J., Nöthen, J., Koch, I.: Reduction techniques for network validation in systems biology. Journal of Theoretical Biology **315**, 71–80 (2012)
3. Lipton, R.J.: The reachability problem requires exponential space. Research report 62, Dept. of Computer Science, Yale University (1976)

Supplementary Figures S2-S7

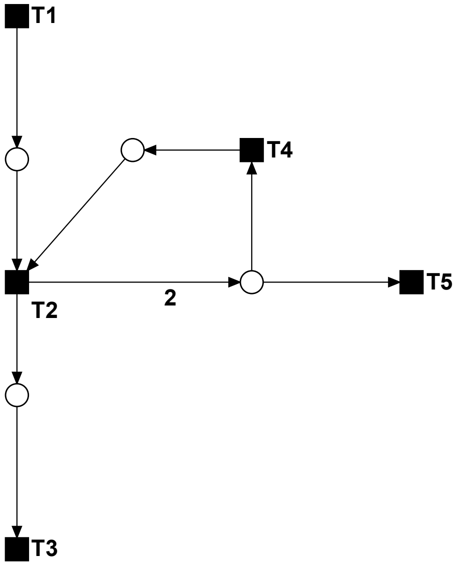

Figure S2 A pure MI of type a. The PN comprises five transitions, four places and nine arcs. It has one TI, $x = (T_1, T_2, T_3, T_4, T_5)$, which consists of all transitions, i.e., it is CTI. The TI is a pure MI, since the network has no PI. Because $T2$ cannot fire in the zero initial marking, the MI is not feasible and, thus, of type a.

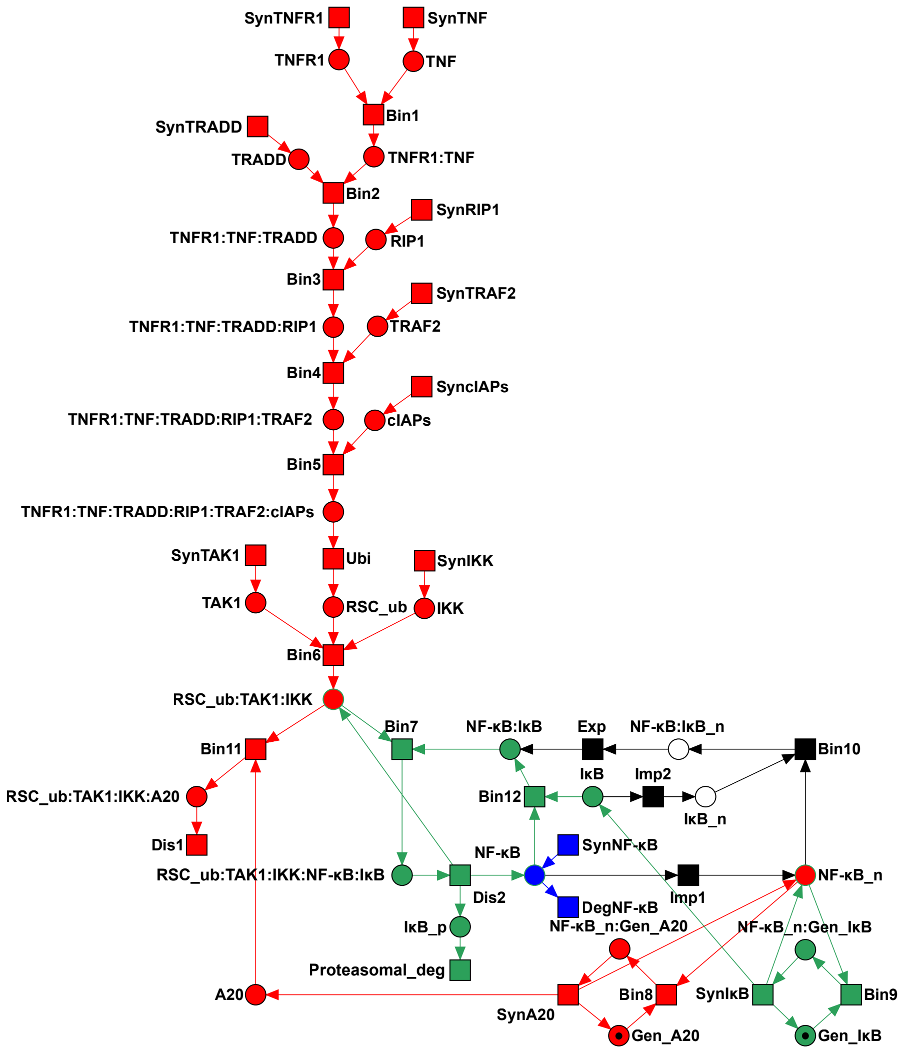

Figure S3 PN of the TNFR1-mediated NF-κB signaling. TI_1 is highlighted blue, TI_2 red and TI_3 green according to Table S4.

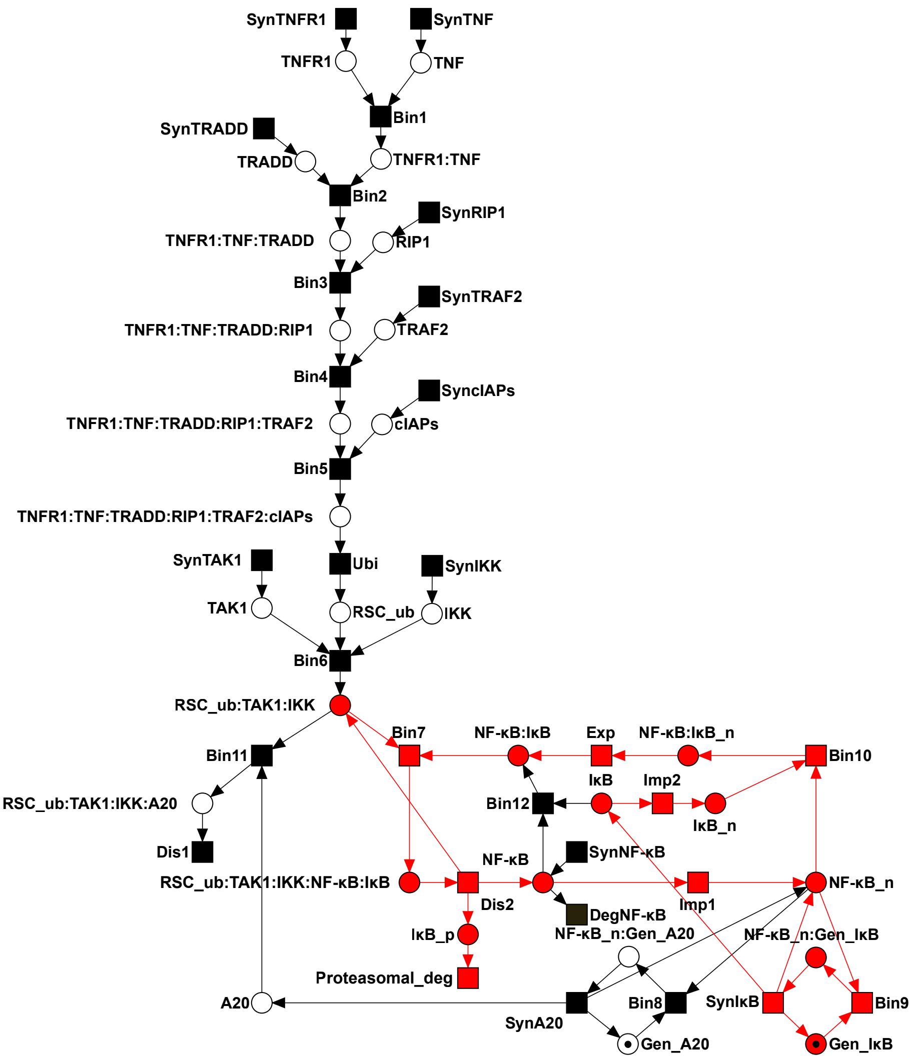

Figure S4 PN of the TNFR1-mediated NF-κB signaling. TI_4 is highlighted red according to Table S4.

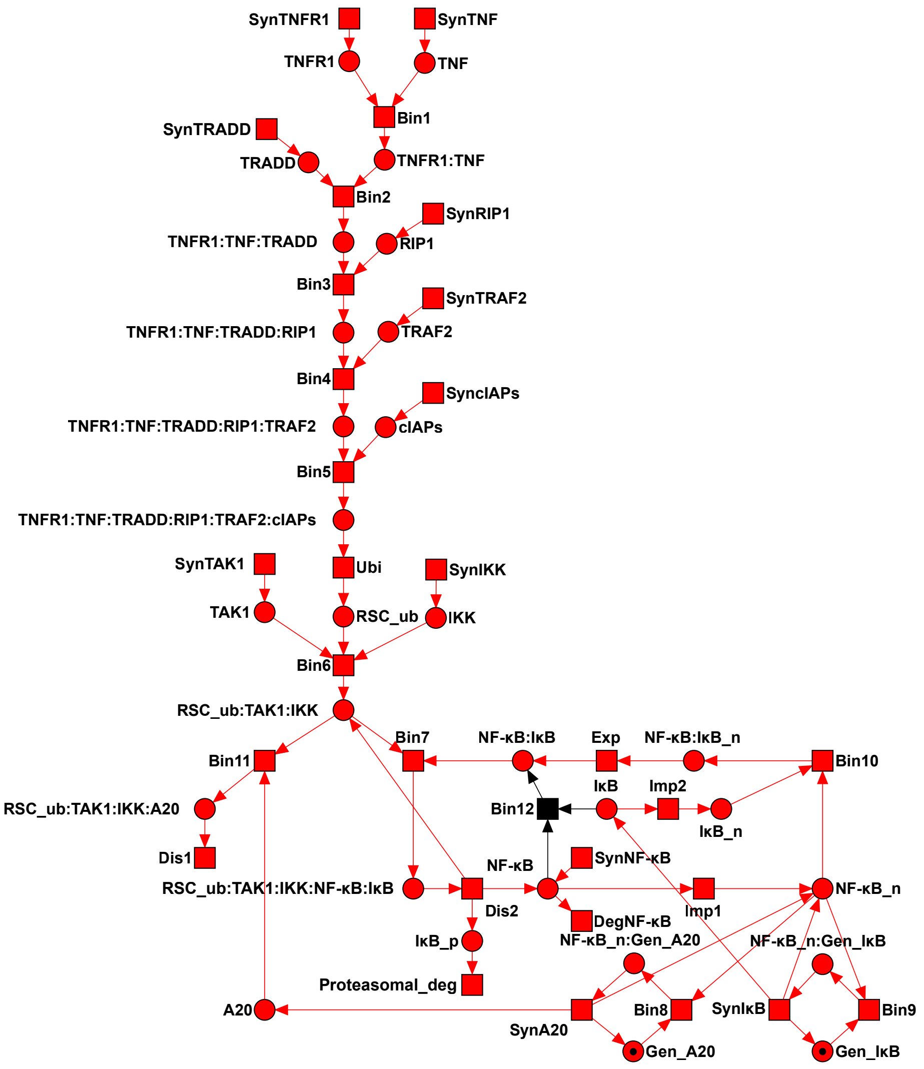

Figure S5 PN of the TNFR1-mediated NF-κB signaling. MI_3 is highlighted red according to Table S5. MI_3 is a linear combination of TI_1, TI_2, and TI_4.

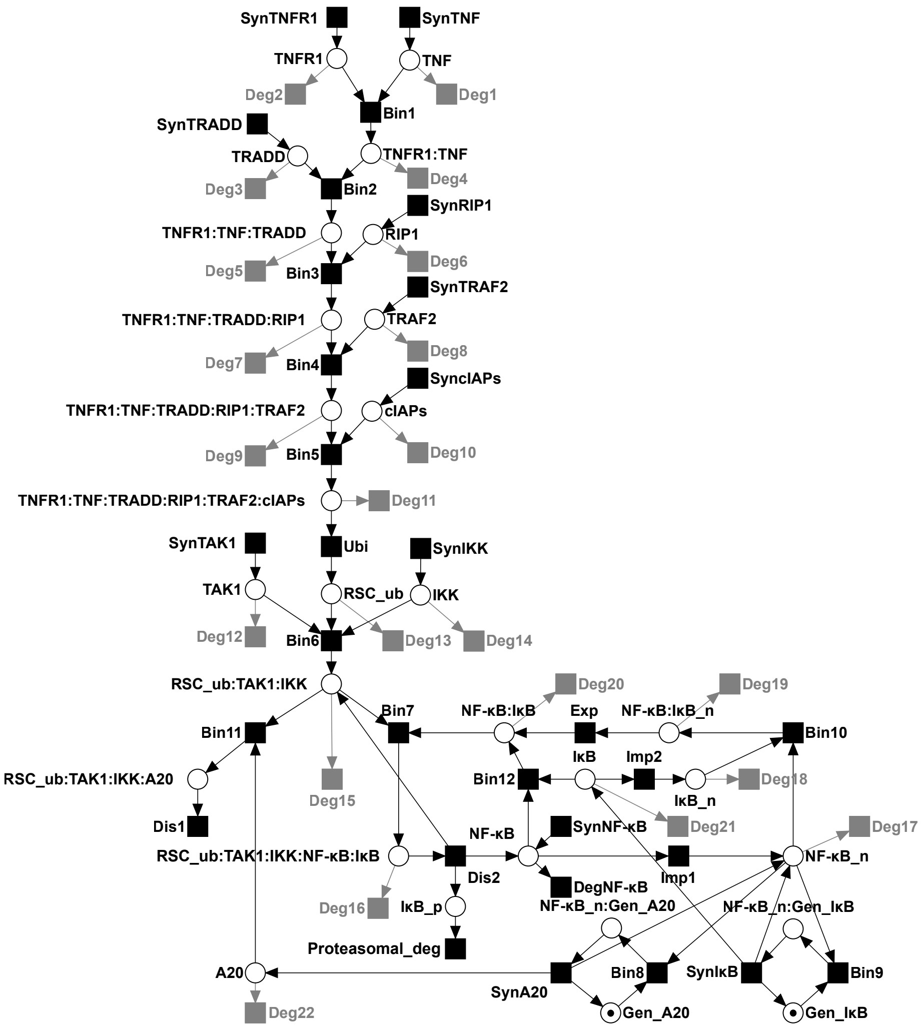

Figure S6 PN of TNFR1-mediated NF-κB signaling with additional output transitions for the *in silico* knockout analysis colored grey.

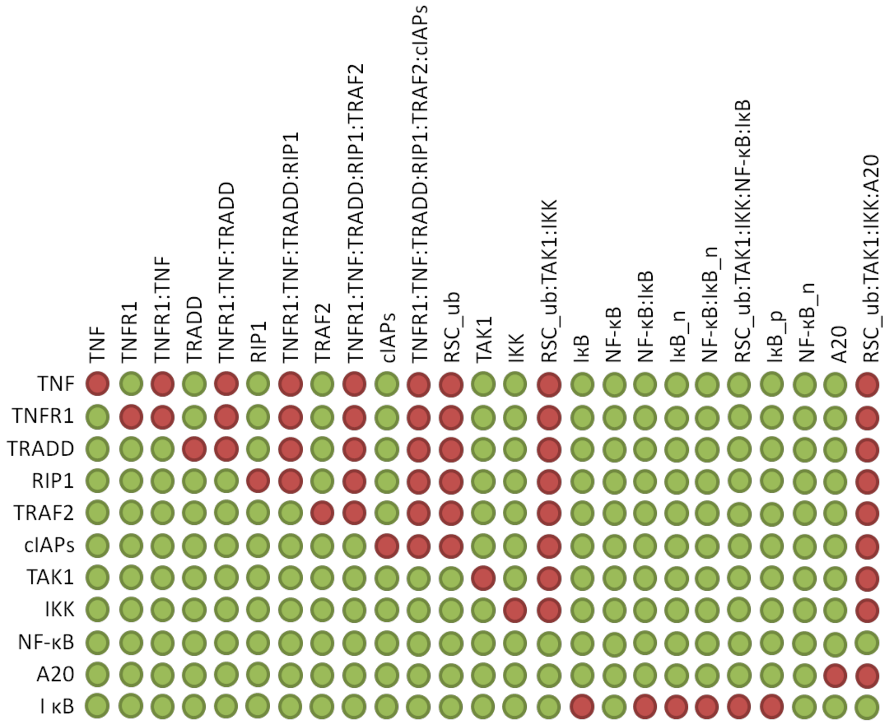

Figure S7 *In silico* knockout matrix based on TI computation for the PN in Figure 3. Proteins that are knocked out are listed in the rows, while the affected proteins and protein complexes are detailed in the columns. The green entries refer to unaffected complexes or proteins in the network, while red entries denote those that are affected by the knockout.

Supplementary Tables S1-S5

Table S1 Places of the PN in Figure 3 and their biological meanings.

Place	Protein/ protein complex
A20	A20 protein
cIAPs	cIAP proteins (cIAP1, cIAP2)
Gen_A20	Gene of A20
Gen_IκB	Gene of IκB
IKK	IKK kinase
IκB	IκB protein
IκB_n	Nuclear IκB protein
IκB_p	Phosphorylated IκB protein
NF-κB	NF-κB protein
NF-κB:IκB	Cytosolic inhibitory complex of NF-κB and IκB
NF-κB:IκB_n	Nuclear inhibitory complex of NF-κB and IκB
NF-κB_n	Nuclear NF-κB protein
NF-κB_n:Gen_A20	Nuclear NF-κB bound to the gene of A20
NF-κB_n:Gen_IκB	Nuclear NF-κB bound to the gene of IκB
RIP1	RIP1 protein
RSC_ub	cIAPs ubiquitinate RIP1 in the RSC
RSC_ub:TAK1:IKK	TAK1 and IKK recruited to the ubiquitinated RSC (TNFR1:TNF-α:TRADD:RIP1:TRAF2:cIAP)
RSC_ub:TAK1:IKK:A20	A20 bound to the ubiquitinated RSC with TAK and IKK
RSC_ub:TAK1:IKK:NF-κB:IκB	RSC with recruited TAK and IKK bound to the inhibitory complex of NF-κB and IκB
TAK1	TAK1 kinase
TNF	TNF-α
TNFR1	TNFR1
TNFR1:TNF	Complex of TNFR1 and TNF-α
TNFR1:TNF:TRADD	Complex of TNFR1, TNF-α and TRADD
TNFR1:TNF:TRADD:RIP1	Complex of TNFR1, TNF-α, TRADD, RIP1
TNFR1:TNF:TRADD:RIP1:TRAF2	Complex of TNFR1, TNF-α, TRADD, RIP1, TRAF2
TNFR1:TNF:TRADD:RIP1:TRAF2:cIAPs	Complex of TNFR1, TNF-α, TRADD, RIP1, TRAF2, cIAPs
TRADD	TRADD protein
TRAF2	TRAF2 protein

Table S2 Transitions of the PN in Figure 3 and additional output transitions (Figure S6) with their biological meanings.

Transition	Biological reaction
Bin1	Binding of TNF-α to TNFR1
Bin2	Binding of TRADD to TNFR1:TNF-α
Bin3	Binding of RIP1 to TNFR1:TNF-α:TRADD
Bin4	Binding of TRAF2 to TNFR1:TNF-α:TRADD:RIP1
Bin5	Binding of cIAPs to TNFR1:TNF-α:TRADD:RIP1:TRAF2
Bin6	Binding of TAK1 and IKK to ubiquitinated RSC
Bin7	Binding of NF-κB:IκB to the ubiquitinated RSC with TAK1 and IKK
Bin8	Binding of nuclear NF-κB to the A20 gene
Bin9	Binding of nuclear NF-κB to the IκB gene
Bin10	Binding of nuclear NF-κB to nuclear IκB
Bin11	Binding of A20 to ubiquitinated RSC with TAK1 and IKK
Bin12	Binding of NF-κB to IκB
DegNF-κB	Degradation of NF-κB
Deg1	Degradation of TNF-α
Deg2	Degradation of TNFR1
Deg3	Degradation of TRADD
Deg4	Degradation of the complex of TNF-α bound to TNFR1
Deg5	Degradation of the complex of TNFR1:TNF-α:TRADD
Deg6	Degradation of RIP1
Deg7	Degradation of the complex of TNFR1:TNF-α:TRADD:RIP1
Deg8	Degradation of TRAF2
Deg9	Degradation of TNFR1:TNF-α:TRADD:RIP1:TRAF2
Deg10	Degradation of cIAPs
Deg11	Degradation of TNFR1:TNF-α:TRADD:RIP1:TRAF2:cIAPs
Deg12	Degradation of TAK1
Deg13	Degradation of ubiquitnated RSC
Deg14	Degradation of IKK
Deg15	Degradation of ubiquitinated RSC with TAK1, IKK
Deg16	Degradation of the complex of NF-κB:IκB bound to the ubiquitinated RSC with TAK1, IKK
Deg17	Degradation of nuclear NF-κB
Deg18	Degradation of nuclear IκB
Deg19	Degradation of the nuclear complex of NF-κB:IκB
Deg20	Degradation of the cytosolic complex of NF-κB:IκB
Deg21	Degradation of IκB
Deg22	Degradation of A20
Dis1	Dissociation of ubiquitinated RSC with TAK1, IKK, A20
Dis2	Dissociation of the complex of NF-κB:IκB bound to the ubiquitinated RSC with TAK1, IKK
Exp	Nuclear export of the inhibitory complex of NF-κB:IκB
Imp1	Nuclear import of NF-κB
Imp2	Nuclear import of IκB
Proteasomal_deg	Proteasomal degradation of IκB
SynA20	Dissociation of the complex of nuclear NF-κB bound to the gene of A20 and synthesis of A20
SyncIAPs	Synthesis of cIAPs
SynIKK	Synthesis of IKK
SynIκB	Dissociation of the complex of nuclear NF-κB bound to the gene of IκB and synthesis of IκB
SynNF-κB	Synthesis of NF-κB
SynRIP1	Synthesis of RIP1
SynTAK1	Synthesis of TAK1
SynTNF	Synthesis of TNF-α
SynTNFR1	Synthesis of TNFR1
SynTRADD	Synthesis of TRADD
SynTRAF2	Synthesis of TRAF2
Ubi	Ubiquitination of RIP1 in the RSC

Table S3 PI of the PN shown in Figure 3.

PI	Places	Meaning
1	NF-κB_n:Gen_A20, Gen_A20	Conservation of the gene of A20
2	NF-κB_n:Gen_IκB, Gen_IκB	Conservation of the gene of IκB

Table S4 TI of the PN, see also Figures S3 and S4.

TI	Transitions	Meaning	feasible
1	SynNF-κB, DegNF-κB	Synthesis and degradation of NF-κB	yes
2	SynTNF, SynTNFR1, Bin1, SynTRADD, Bin2, SynRIP1, Bin3, SynTRAF2, Bin4, SyncIAPs, Bin5, Ubi, SynTAK1, SynIKK, Bin6, Bin8, SynA20, Bin11, Dis1	TNFR1 activation and RSC formation and dissociation induced by A20 gene expression	no
3	Bin7, SynIκB, Bin9, Dis2, Proteasomal deg, Bin12	IκB expression and formation of the inhibitory complex with NF-κB and subsequent dissociation of the complex and activation of NF-κB by *RSC_ub:TAK1:IKK*	no
4	Bin7, SynIκB, Imp2, Bin9, Exp, Bin10, Dis2, Proteasomal deg, Imp1	Regulation of NF-κB activity, i.e., degradation of the inhibitory complex of IκB and NF-κB, NF-κB-dependent initiation of IκB gene expression, restoration of the inhibitor in the cytosol, formation of the inhibitory complex in the nucleus and translocation of the inhibitory complex of NF-κB and IκB into the cytosol	no

Table S5 MI of the PN. MI_1 is identical to TI_1, MI_2 covers the complete PN and MI_3 is depicted in Figure S5.

MI	Linear combination of TI	Meaning	feasible
1	1	Synthesis and degradation of NF-κB	yes
2	1,2,3,4	TNFR1-mediated NF-κB signaling pathway, i.e., from receptor ligation to NF-κB activation and initiation of terminating feedback loops	yes
3	1,2,4	TNFR1-mediated NF-κB signaling pathway, i.e., similar to MI_2, but the formation process of the inhibitory complex of NF-κB and IκB differs	yes
